# Revealing Solid Properties of High-energy-density Molecular Cocrystals from the Cooperation of Hydrogen Bonding and Molecular Polarizability

**DOI:** 10.1038/s41598-018-37500-y

**Published:** 2019-02-04

**Authors:** Lei Zhang, Sheng-Li Jiang, Yi Yu, Jun Chen

**Affiliations:** 1 Software Centre for High Performance Numerical Simulation, Institute of Applied Physics and Computational Mathematics, Beijing, 100088 People’s Republic of China; 2 Laboratory of Computational Physics, Institute of Applied Physics and Computational Mathematics, Beijing, 100088 People’s Republic of China; 3 Center for Applied Physics and Technology, Peking University, Beijing, 100871 China

## Abstract

In the domain of high-energy-density materials, the understanding to physico-chemical properties has long been primarily based on molecular structures whereas the crystal packing effect that significantly affects solid properties has been seldom involved. Herewith we predict the solid properties of six novel energetic cocrystals by taking into account of the crystal packing effect using a quantum chemistry method. We discover that the hydrogen bonding causes an increase in the molecular polarizability and their cooperation significantly changes the solid-state nature of the cocrystals compared to the pristine crystal and the gas counterparts. For example, stabilizing the multi-component molecular association by increasing the binding energy by 19–41% over the pristine crystals, improving the detonation performance by 5–10% and reducing the sensitivity to external stimuli compared to their pure crystal or gas counterparts. Therefore, the solid nature of the cocrystal is not a simple combination of the pure crystalline properties of its components and the heterogeneous molecular coupling effects must be considered to design improved functional cocrystals.

## Introduction

Cocrystallization, tailoring crystal packing effects by intermolecular charge transfer and intramolecular charge redistribution, has been supposed to be an effective way to tune the nature of functional materials over their pristine counterparts^1–6^. In designing improved high-energy-density materials, a key goal is to achieve an optimum balance between two inherently contradictory objectives: a high level of detonation performance and low sensitivity to accidental initiation of detonation^7,8^. The introduction of cocrystallization technology into energetic materials domain has given us hope to break the dilemma^1,9,10^. For example, using high-energy-density hexanitrohexaazaisowurtzitane molecule (CL-20, C_6_H_6_N_12_O_12_) and low-sensitivity octahydro-1,3,5,7-tetranitro-1,3,5,7-tetrazocine molecule (HMX, C_4_H_8_N_8_O_8_) as raw materials, the obtained 2:1 CL-20:HMX cocrystal has a firing power close to CL-20^11^ and similar safety properties to HMX. By mixing CL-20 molecules and low-sensitivity 2,4,6-trinitrotoluene molecules (TNT, C_7_H_5_N_3_O_6_), 1:1 CL-20:TNT cocrystal has twice the stability of CL-20 and is safe enough to transport^12^.

To effectively achieve the engineering of improved energetic cocrystals, the critical issues are how the multicomponent molecules interact to stabilize the crystal structure and tune the solid nature. However, from years the quantum chemical understanding to physicochemical properties of high-energy-density crystals have been based solely on molecular structures^13–16^. Most of the researches were limited to the development of empirical quantitative structure-property relationships (QSPR) model based on the constitutional, topological, geometric, or quantum chemical descriptions of free molecules^8,17,18^. The crystal packing effect, although important and decisive to the solid properties, has been seldom involved^8,19–21^.

To this end, we study the solid properties of six novel high-energy-density molecular cocrystals composed of benzotrifuroxan (BTF, C_6_N_6_O_6_)^22,23^, nitro compound molecules including 2,4,6-trinitrobenzene methylamine (MATNB, C_7_N_4_O_6_O_6_), 2,4,6-trinitroaniline (TNA, C_6_N_4_O_6_O_4_), 1,3,3-trinitroazetidine (TNAZ, C_3_N_4_O_6_O_4_), 1,3,5-Trinitrobenzene (TNB, C_6_N_3_O_6_O_3_), TNT, and nitroamine molecule CL-20. BTF is one of the most powerful explosives available in the commercial field currently, but it is sensitive to external stimuli. In the impact tests, the measured *h*_50%_ of BTF (21 *cm*^22^ or 50 *cm*^8^) was significantly smaller than that of the insensitive explosive triaminotrinitrobenzene (TATB) (>320 *cm*^8^). Introducing hydrogen-containing component to the BTF molecules is expected to provide improved overall properties of the novel cocrystals, however, most of the properties cannot be obtained experimentally, because in addition to safety issues, the sample amount of the novel product is difficult to up to standard.

Herewith, using a quantum chemistry method, we predict the structural, thermodynamic, explosion and safety properties of a series of typical novel energetic cocrystals and evaluate the multicomponent molecular packing effect. An interesting coupling of hydrogen bonding and molecular polarizability and their effect in tuning the solid-state nature of the cocrystals is reported. Our work is expected to provide useful reference towards designing improved energetic-energetic cocrystals.

## Results and Discussion

### Repulsive inter-lone-pair interactions and Attractive proton-lone pair interaction

**Figure 1:**
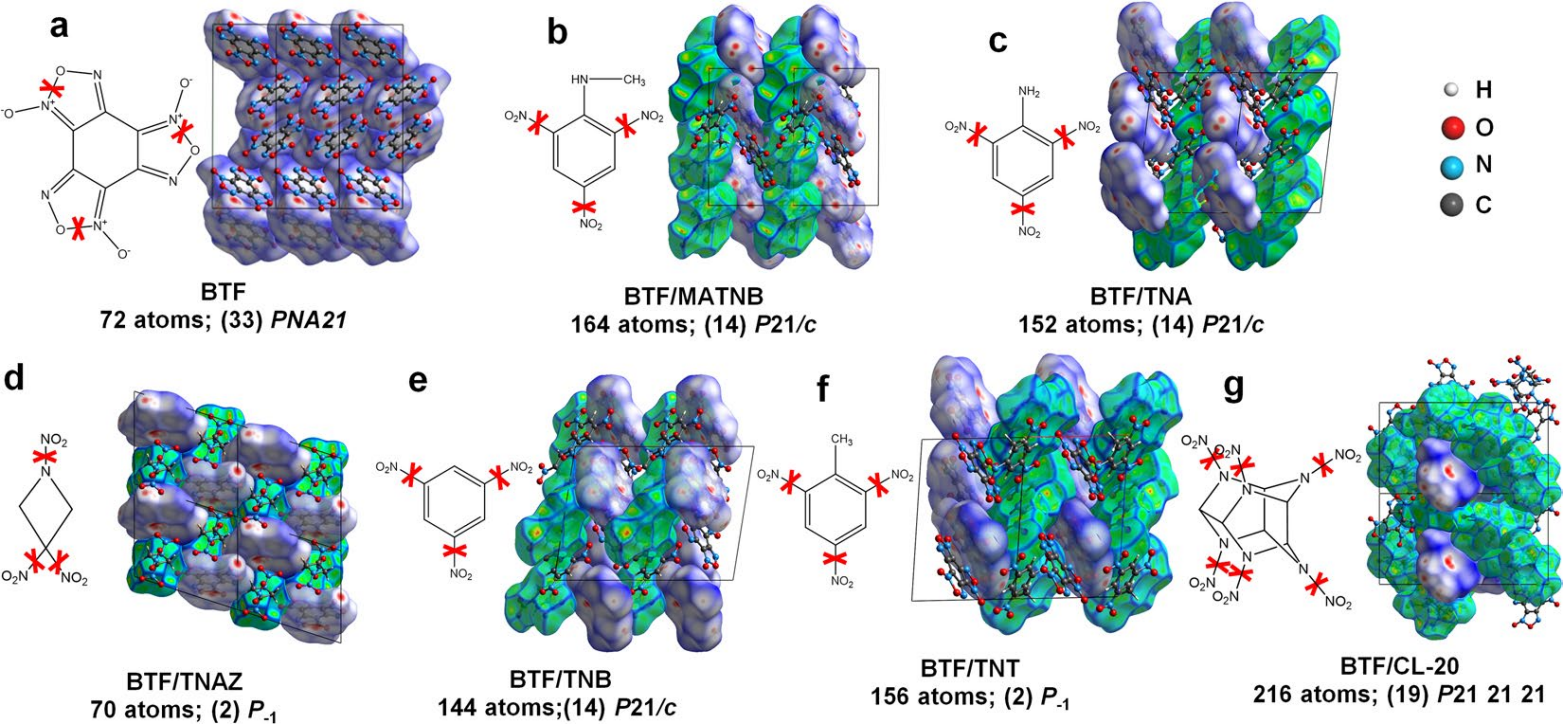
Chemical diagrams of high-energy-density molecules of BTF, MATNB, TNA, TNAZ, TNB, TNT, and CL-20; 3D crystal packing structures of pristine BTF crystal and BTF/MATNB, BTF/TNA, BTF/TNAZ, BTF/TNB, BTF/TNT, and BTF/CL-20 cocrystals. The domain of each individual molecule is illustrated by the corresponding Hirshfeld surface: the BTF molecule is enclosed by blue surfaces and the other molecules are enclosed by the green. The red-crosses denote the trigger linkages for chemical reaction initiation for each molecule.

**Figure 2:**
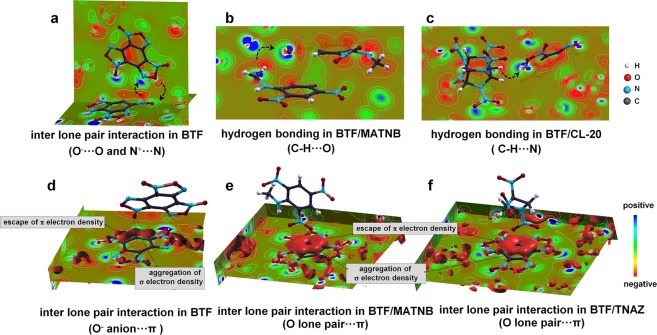
Intermolecular charge transfers due to crystal packing effect in (**a**) pristine BTF crystal, (**b**) BTF/MATNB cocrystal, and (**c**) BTF/CL-20 cocrystal. Escape of π electron density and aggregation of σ electron density at the benzene ring backbone in (**d**) pristine BTF crystal, (**e**) BTF/MATNB cocrystal, and (**f**) BTF/TNAZ cocrystal. The π electron density escape is illustrated by the isosurfaces for both pristine BTF crystal (−0.4 *e*/nm^3^) and cocrystals (−0.7 *e*/nm^3^).

Intermolecular charge transfer and intramolecular charge redistribution are observed in the structures of BTF, BTF/MATNB, BTF/TNA, BTF/TNAZ, BTF/TNB, BTF/TNT, and BTF/CL-20 due to the periodic packing of molecules in space, as shown in Figs 1 and 2. Such molecular packing introduces three types of intermolecular interactions for the close-confronting atoms – inter-lone-pair interactions, lone-pair-π interactions and proton-lone pair interactions (*i*.*e*. hydrogen bonding)^24–26^.

BTF molecule consists of electronegative elements of oxygen and nitrogen, which have two pairs and one pair of lone-pair electrons, respectively. Thus, all the interactions within BTF crystal are inter-lone-pair interactions in addition to the aromatic stacking interactions (Fig. 2). According to the calculated crystal orbital Hamilton population (COHP), all the inter-lone-pair interactions (N···N, O···N, and O···O) and lone pair-π interactions (O···C) are antibonding and furnish each a positive item to the total energy, ranging from +0.69 *kcal/mol* to +1.15 *kcal/mol* (Table 1 and Supplementary Fig. S2). Therefore, in the equilibrium crystal structure, such inter-lone-pair  and lone-pair-π  interactions are mutually exclusive and serve to balance the *van der Waals* attraction items.

**Table 1:** Distances (*D*, in Å) and strengths (*S*, in *kcal/mol*) of the intermolecular interactions due to crystal packing effect.

Types		BTF crystal		BTF/MATNB		BTF/TNA		BTF/TNAZ		BTF/TNB		BTF/TNT		BTF/CL-20	
		*D*	*S*	*D*	*S*	*D*	*S*	*D*	*S*	*D*	*S*	*D*	*S*	*D*	*S*
BTF-BTF	N···N	2.98	+1.15	3.06	+1.84	3.01	+1.61	3.26	+0.23	3.12	+1.38	2.95	+2.30	—	—
	O···C	2.99	+0.69	—	—	3.09	+0.69	—	—	3.09	+0.46	3.11	+0.69	2.84	+1.15
	O···O	2.93	+0.69	2.86	+1.38	—	—	3.02	+1.84	3.02	+0.69	3.07	+0.69	—	—
	O···N	3.03	+1.61	3.10	+0.92	3.06	+0.69	3.10	+0.69	3.04	+1.15	3.01	+0.92	—	—
BTF-Coformer	**H···O**	−	−	**2.42**	**−1.38**	**2.40**	**−1.61**	**2.43**	**−0.92**	**2.57**	**−1.15**	**2.54**	**−1.15**	**2.47**	**+0.23**
	**H···N**	—	—	—	—	—	—	—	—	—	—	**2.58**	**+0.69**	**2.47**	**−3.22**
	O···C	—	—	3.02	+0.46	2.93	+1.15	2.67	+0.69	2.98	+0.46	—	—	3.01	+0.69
	O···O	—	—	2.86	+1.61	2.87	+1.15	2.77	+1.15	—	—	—	—	2.81	+1.61
	O···N	—	—	—	—	2.96	+0.23	2.66	+0.23	2.89	+1.38	2.88	+0.23	2.89	+1.38
Coformer-Coformer	**H···O**	—	—	**2.49**	**−2.53**	**2.59**	**−0.46**	—	—	**2.36**	**−1.38**	**2.59**	**−0.92**	**2.44**	**−1.38**
	O···C	—	—	—	—	—	—	—	—	3.08	+0.69	3.08	+1.15	3.03	+0.92
	O···O	—	—	—	—	—	—	—	—	2.98	+0.92	—	—	2.91	+0.69
	O···N	—	—	2.91	+0.69	—	—	—	—	—	—	2.90	+0.23	2.94	+0.23

The hydrogen bonding interactions are marked by the bold. The information of covalent bonds is given in supplementary Table S2. The hydrogen bonding interactions are marked by the bold. The information of covalent bonds is given in supplementary Table S2.

In BTF/MATNB, BTF/TNA, BTF/TNAZ, BTF/TNB, BTF/TNT, and BTF/CL-20 cocrystals, the proton-lone pair interactions, like C-H···O and C-H···N, are present in addition to the inter-lone-pair and lone-pair-π  interactions. The proton-lone pair interactions occupy 18.6~28.7% of entire intermolecular charge population. These hydrogen bonds are 2.36~2.59 Å long and the hydrogen bond energies are up to 3.22 *kcal/mol*. As compared to the inter-lone-pair  and lone-pair-π interactions, the proton-lone pair distances are roughly 20% shorter and the interactions are all attractive (Table 1). Therefore, these hydrogen bonding interactions play an indispensable role in reducing the total energy, propelling the combination of multi-component molecules into cocrystals.

### Cooperation of the intermolecular interactions and the molecular polarizability

Interestingly, we find that the inter-lone-pair  and lone-pair-π interactions can make the non-polar molecule polar, as shown in Table 2 and Fig. 2. In BTF crystal, the periodically distributed interactions break the original C_*3h*_ symmetry of the charge distribution in a free BTF molecule. The Mulliken population of the carbon atoms in the benzene ring decreases from 3.83 *e* to 3.81 *e* and that around the O atom in the at the border of the oxofurazan ring increases from 6.46 *e* to 6.49 *e*. Therefore, two thirds of the three N^+^-O^−^ bonds become more polar whereas the left one third less polar. By this way, the total dipole moment of the BTF molecule increased to ~0.3 *Debye*.

**Table 2:** Enhancements of molecular polarizabilities (in *Debye*) due to multicomponent molecular packing effect in the seven studied systems.

	Isolate	BTF crystal	BTF/MATNB	BTF/TNA	BTF/TNAZ	BTF/TNB	BTF/TNT	BTF/CL-20
BTF	0.0	0.3 (↑)	0.7 (↑)	0.9 (↑)	0.2 (↑)	1.2 (↑)	0.8 (↑)	0.7 (↑)
MATNB	5.2		6.8 (↑)					
TNA	2.4			3.6 (↑)				
TNAZ	1.6				1.6			
TNB	0.0					0.7 (↑)		
TNT	2.0						3.6 (↑)	
CL-20	0.9							2.4 (↑)

Due to the greater distinction of the electronegativity of hydrogen (2.2) from other element (oxygen: 3.5, nitrogen: 3.0, carbon: 2.6), the proton-lone pair interactions generally provoke doubled amount of charge transfer of inter-lone-pair interactions, and thus cause more increase in the molecular polarizability (Table 2 and Fig. 2). Furthermore, the presence of more electronegative nitro groups (than amine oxide group) exacerbates the escape of π electron density from the BTF molecules. By such way, the intramolecular charge distributions in the cocrystals become more anisotropic so the molecules are more polarized as compared to the pristine BTF crystal. As shown in Table 2, the dipole moment increase in the molecules of the cocrystals are between 0.7 and 1.2 *Debye*, more than in the BTF crystal (~0.3 *Debye*). Also, the TNB molecule has no dipole at the free state due to its D_*3h*_ symmetry, but its dipole moment increases to 0.7 *Debye* when it is present in the BTF/TNB cocrystal.

### Solid-state properties of the cocrystals: thermodynamics, structure, explosion and safety

**Figure 3:**
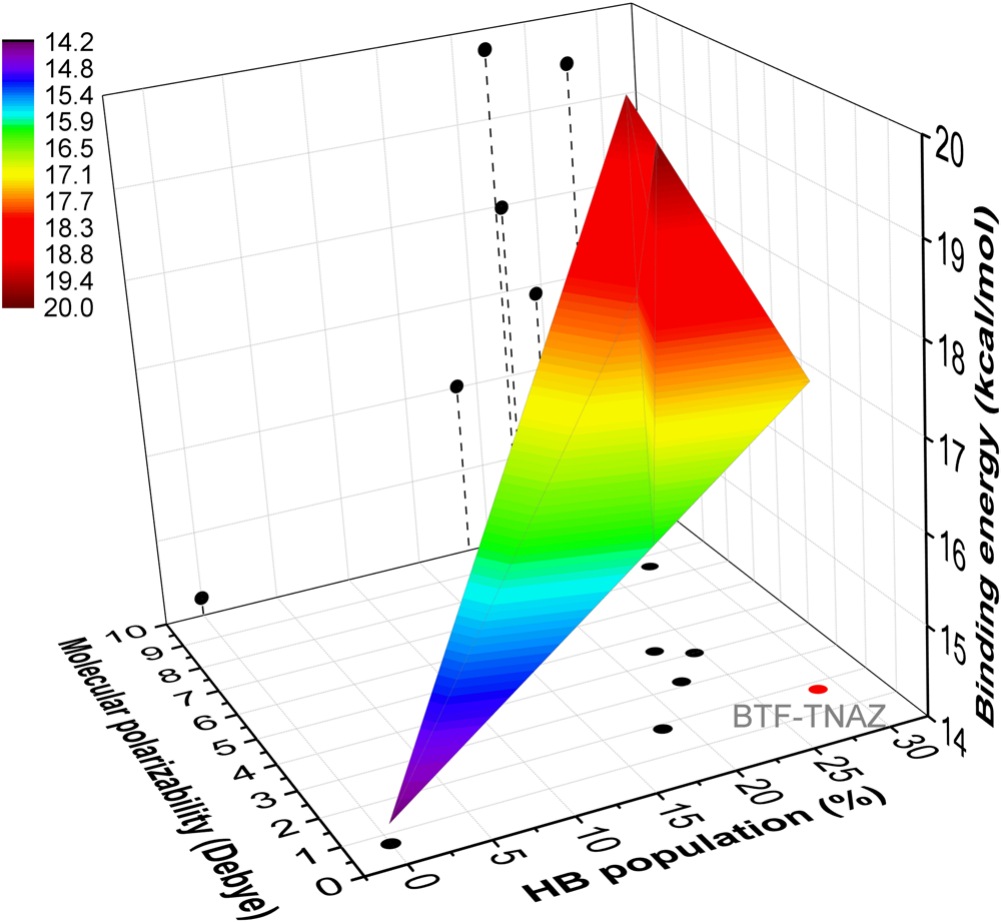
Cooperation of hydrogen bonding and molecular polarizability in increasing intermolecular binding energies (in *kcal/mol*) of the multiple-component molecular associations. The contribution of hydrogen bonding is represented by its charge proportion occupied in the Hirshfeld surfaces.

The cooperation of the hydrogen bonding and the molecular polarizabilities increases the binding energies of the cocrystals by 19–41% as compared to the pure BTF crystal, as shown in Fig. 3 and Table 3. The increase of the binding energies implies that the multicomponent association is thermodynamically more stable as compared to the pristine crystal.

**Table 3:** Predicted solid-state properties of the seven systems, including binding energies (*kcal/mol*), crystal structure characters (including packing coefficient, in % and density, in *g/m*^3^), chemical composition (oxygen balance, in %), detonation performance (heat of explosion, in *kcal/kg*, explosion temperature, in *K*, velocity of detonation, in *km/s*, and detonation pressure, in *GPa*).

	BTF	BTF/MATNB	BTF/TNA	BTF/TNAZ	BTF/TNB	BTF/TNT	BTF/CL-20
Binding energy	14.21	19.46	17.98	17.36	16.01	16.90	19.77
Packing coefficient	72.02	72.34	73.55	75.10	73.21	73.21	72.39
Density	1.85 (1.90^32^,1.85^33^)	1.75 (1.80^22^)	1.82 (1.88^22^)	1.84 (1.83^22^)	1.79 (1.81^22^)	1.75 (1.81^22^)	1.85 (1.92^25^)
Oxygen balance	−38.08	−55.03	−46.64	−28.81	−46.43	−55.08	−20.86
Heat of explosion	1474 (1410^33^)	1325	1322	1492	1412	1389	1430
Explosion temperature	5268 (3700-5570^34–36^)(3700-5570^34–36^)5268 (3700-5570^34–36^)(3700-5570^34–36^)	4131	4336	4888	4638	4335	4844
Detonation pressure	29.78 (35.1^33^, 36^37^)	25.04	27.34	32.35	26.94	25.21	33.31
Velocity of detonation	8.05 (8.49^33,37^)	7.51	7.76	8.40	7.73	7.53	8.51

The properties of the pristine BTF crystal from experiments and other calculations are tabulated for comparison (in brackets).The properties of the pristine BTF crystal from experiments and other calculations are tabulated for comparison (in brackets).

From the structural point of view, the packing coefficient (PC) of a solid is determined by the competition of two factors: compatibility and intermolecular binding energy. Similar polarizability is known to increase the solubility of two types of ingredient molecules. The pristine BTF crystal has the lowest PC of 72.02% because its binding energy is the lowest, at 14.21 kcal/mol. Whereas, for BTF/CL-20 cocrystal, although the binding energy is up to 19.77 kcal/mol, the large difference between the polarizabilities of BTF molecule and CL-20 molecule cuts off the intermolecular compatibility in crystal packing [Supplementary Fig. S3], leading to a smaller PC of 72.39%.

Our calculations indicate that the detonation performances of the cocrystals are altered over the pristine crystals due to the inter-multicomponent coupling interactions. Bulk density is a recognized criterion of detonation performances of high-energy-density substances. Although the introduction of hydrogen element in the cocrystals benefits intermolecular attractions, thus helping to denser molecular packing in space, but it is not conducive to the improvement of bulk density due to its small mass. Therefore, the bulk density of hydrogen-free BTF crystal and those of the hydrogen-including cocrystals of BTF/MATNB, BTF/TNA, BTF/TNAZ, BTF/TNB, and BTF/TNT are similar. For the detonation performance of pure BTF crystal, the heat of explosion, explosion temperature, velocity of detonation, and detonation pressure are 1474 *kcal/kg*, 5268 *K*, 8.05* km/s*, and 29.78 *GPa*, respectively, showing satisfactory agreement with the experiments, as shown in Table 3. The BTF/CL-20 cocrystal owns similar bulk density but much improved oxygen balance as compared to pure BTF solid. Therefore, its detonation performances are significantly ameliorated, with the velocity of detonation improved by ~10% and detonation pressure improved by ~5%, respectively (Table 3).

Another multicomponent molecular packing effect is the reduced sensitivity to external stimuli of these energetic cocrystals, manifested in the strengthening of all the chemical reaction trigger linkages as compared to their pure crystal and gas counterparts (Table S1). Here the assumption is that all the absorbed energies from external stimuli are devoted to alter the vibrational modes of the weakest covalent bonds.

For BTF crystal, the chemical reaction trigger linkages are the three N^+^-O covalent bonds in the oxofurazan ring and their strengths are 2.53 *kcal/mol*, 3.22 *kcal/mol*, and 4.14 *kcal/mol*, respectively, higher than the gaseous BTF molecule (Supplementary Table S1 and Fig. S4). Such strengthening effect is proved to be more pronounced in the hydrogen-including systems. As shown in Table 4, the bond strengths increase by 2.07~8.28* kcal/mol* for N-NO_2_ and by 7.13~10.35 *kcal/mol* for C-NO_2_.

**Table 4:** Enhanced strengths (*kcal/mol*) of the trigger linkages of chemical reaction initiation as compared to their counterparts in gas.

	Isolate	BTF	BTF/MATNB	BTF/TNA	BTF/TNAZ	BTF/TNB	BTF/TNT	BTF/CL-20
BTF (N^+^-O)	−42.09	−45.31(↑)	−46.92(↑)	−46.23(↑)	−43.70(↑)	−45.77(↑)	−45.54(↑)	−45.54(↑)
MATNB (C-NO_2_)	−101.89		−110.17(↑)					
TNA (C-NO_2_)	−103.96			−106.72(↑)				
TNAZ (N-NO_2_)	−99.36				−109.71(↑)			
TNAZ (C-NO_2_)	−91.77				−93.84(↑)			
TNB (C-NO_2_)	−97.52					−99.82(↑)		
TNT (C-NO_2_)	−97.29						−100.74(↑)	
CL-20 (N-NO_2_)	−102.81							−109.94(↑)
*h*_50%_		21^22^	<17.8^22^	<17.8^22^	<12.6^22^	42.2^22^	36.2^22^	—

The trigger linkages are N^+^-O covalent bonds in the oxofurazan ring of BTF molecule, C-NO_2_ bonds in nitro compound molecules, and N-NO_2_ bonds in nitroamine molecules, and they are denoted by red crosses in Fig. 1. The *h*_50%_ (in *cm*) of these crystals obtained in the impact tests are as listed for comparison.The trigger linkages are N^+^-O covalent bonds in the oxofurazan ring of BTF molecule, C-NO_2_ bonds in nitro compound molecules, and N-NO_2_ bonds in nitroamine molecules, and they are denoted by red crosses in Fig. 1. The *h*_50%_ (in *cm*) of these crystals obtained in the impact tests are as listed for comparison.

For BTF/TNB and BTF/TNT cocrystals, the enhancement of the trigger linkages over the pure BTF crystal (by 0.46* kcal/mol* and 0.23* kcal/mol*, respectively) indicates their reduced sensitivities, well confirmed by the measured *h*_50%_ (21 *cm* for BTF, 42.2* cm* for BTF/TNB and 36.2 cm for BTF/TNT^22^) as shown in Table 4. This result implies that the solid nature of the cocrystal is not a simple combination of the pure crystalline properties of its constituents. Nonlinear effects like the coupling of the hydrogen bonding and molecular polarizability may give the cocrystal improved sensitivity than any of its pure compounds. Whereas, the inconsistent sensitivity prediction between the calculation and the experiments for the BTF/MATNB and BTF/TNA cocrystals warns us that accurate assessment of the inherent sensitivity of single crystal (defect free), from both experimental and computational perspectives, remains a challenging task to be explored.

To conclude, we have predicted the solid nature of six novel energetic cocrystals using quantum chemical method and discovered an interesting coupling of hydrogen bonding and molecular polarizability. Their cooperation significantly enhances the stability of the multicomponent molecular association, alters the crystal packing characteristics and improves the detonation performance and the safety of the cocrystal solid as compared to their pristine compounds. Therefore, in order to rationally design improved energetic cocrystals, the heterogeneous molecular coupling effects must be taken into account.

### Methodology

#### General information

All the calculations were performed using the recently developed density functional theory (DFT) HASEM package^27,28^. The generalized gradient approximation was used for the exchange-correlation functional in the Perdew-Burke-Ernzerhof form. Norm-conserving pseudopotentials specialized for high-energy-density molecular crystals were used to replace the core electrons. The valence electrons were described by linear combinations of numerical pseudoatomic orbitals. The reliability of this method to predict the intermolecular interaction energies and to predict the nature of high-energy-density molecular crystals has been confirmed in the previous work^27,29–31^.

#### Structural optimization

Taking the information obtained from X-ray diffraction analysis as input^22,23,32^, the geometry optimizations for the crystal lattices and atomic positions of BTF, BTF/MATNB, BTF/TNA, BTF/TNAZ, BTF/TNB, BTF/TNT, and BTF/CL-20 cocrystals were performed on the basis of conjugate gradient method (Fig. 1). The simulated structures were considered as finally optimized when the stress components were less than 0.01 GPa and the residual forces were less than 0.03 eV/Å. For all the studied seven systems, the calculated structures showed satisfactory agreement with the experiments: the discrepancies of the lattice parameters ranged from −2.03% to +2.82% and that of the volumes ranged from −0.51% to 3.74% (Supplementary Fig. S1).

#### Characterization of solid nature

Based on the optimized structures of the seven systems, the intermolecular charge transfers, inter-/intra-molecular interaction strengths, molecular polarizabilities, bulk density, packing coefficient, and binding energies were calculated to characterize the crystal packing effect. The detailed calculation methods are provided in the supporting information. The predicted macro solid properties include detonation performances and sensitivities to accidental initiation of detonation. For the prediction of the detonation performance, we used a recently developed method by taking into account of the statistical correction from experimental data, which were detailed in our previous work^29^ (Supplementary Fig. S5). The nature of BTF has already been clarified by plenty of experimental tests and simulations and these acquired data are in turn used to confirm the reliability of the current predictions.

## Supplementary information

supplementary information

supplementary information

## Data Availability

The source data that support the plots in this Article and the other findings of this study are available from the corresponding authors upon reasonable request.
