# Supplementary material for: Revealing Solid Properties of High-energy-density Molecular Cocrystals from the Cooperation of Hydrogen Bonding and Molecular Polarizability
Source: Sci Rep. 2019 Feb 4;9:1257. doi: 10.1038/s41598-018-37500-y (PMC6362133; doi:10.1038/s41598-018-37500-y)
Supplement: Supplementary file 1 — supplementary information [file 41598_2018_37500_MOESM1_ESM.pdf]

Supplementary Materials for

# Revealing Solid Properties of High-energy-density Molecular Cocrystals from the Cooperation of Hydrogen Bonding and Molecular Polarizability

*Lei Zhang[a,b*], Sheng-Li Jiang[a*], Yi Yu[a], and Jun Chen[a,b,c†]*

[a.] *Software Centre for High Performance Numerical Simulation, Institute of Applied Physics and Computational Mathematics, Beijing, 100088, People's Republic of China.*

[b.] *Laboratory of Computational Physics, Institute of Applied Physics and Computational Mathematics, Beijing, 100088, People's Republic of China.*

[c.] *Center for Applied Physics and Technology, Peking University, Beijing, 100871, China.*

*These authors contributed equally to this work

†Corresponding author. E-mail: Jun_chen@iapcm.ac.cn

**This PDF file includes:**

S1. Characterization methods of crystal packing effects

Figs. 1S to 5S

Tables 1S to 2S

## S1. Characterization methods of crystal packing effects

The intermolecular charge transfer was characterized by visualizing the charge density difference and calculating the Mulliken electron population. Charge density difference was obtained by subtracting the density values of each ingredient molecule from the total density values of the system. Therefore the charge distribution surrounding each individual molecule should be calculated in advance. When calculating the charge density of one molecule in BTF crystal, the other molecules were removed but the ghost orbitals remained. Then, the interactions among non-equivalent molecules could be clearly tracked by looking at the charge density difference. Mulliken population was employed to quantify the local charge distribution surrounding an atom at the border of the molecules.

The molecular polarizability was characterized by its dipole moment. The molecular dipole moment was calculated by the distance of the positive charge centroid and the negative charge centroid of the molecule timed the total electron of the molecule. Electrical neutrality of the molecule was required. Here we have made an approximation that the charge distributions of the atoms were spherical. Therefore, the estimated molecular dipole moment was slightly higher than a true value. For a more accurate value of dipole moment, an integration of charge distribution over the entire space was needed.

Using CrystalExplorer3.1[1], we plotted Hirshfeld surfaces to discern intermolecular interaction types and to visualize the spatial packing configuration of constituent molecules. In order to evaluate the contribution of hydrogen bonding to the stabilizing nature of the cocrystals, we simply used the quantity of hydrogen bonding population on the enclosed Hirshfeld surface of the coformer molecule. We note this was qualitative because sometimes, although the hydrogen bonding strength was weak, it still occupied a large percentage of area in the Hirshfeld surface of a molecule, like the case of BTF/TNAZ cocrystal.

For the local interatomic contacts, both intermolecular close contacts and intramolecular covalent bonds, the interactive strength was quantified by the integrated value of crystal orbital Hamilton population (COHP). The COHP resulted from multiplying the electron density of states (DOS) by the overlap element of the Hamiltonian. It partitioned the band-structure energy into orbital-pair interactions and involved the bonding information: negative values identified bonding interactions and positive values identified antibonding interactions. COHP showed the contribution of a specific interatomic contact to the band energy and the integrated COHP hinted towards the interactive strength.

The intermolecular binding energy was defined as the difference between total energy of constituent ions in the free state and total energy of the crystal. The total energy of a free molecule was calculated by putting one molecule in vacuum modeled by a $30\times30\times30$ Å$^3$ cubic. Positive binding energy means that converting from gas to solid is energetically preferable and vice versa.

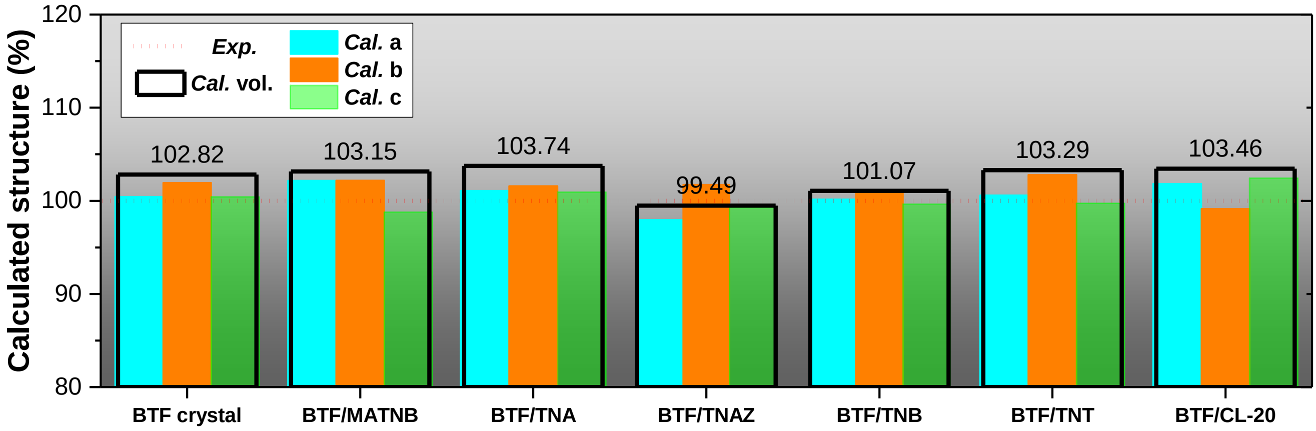


**Figure 1S** (Color online) Lattice parameters for all seven systems: DFT results and experimental measurements. The calculated structures and the experiments are satisfactorily consistent, with the discrepancies of the lattice parameters ranged from -2.03% to +2.82% and that of the volumes ranged from -0.51% to 3.74%

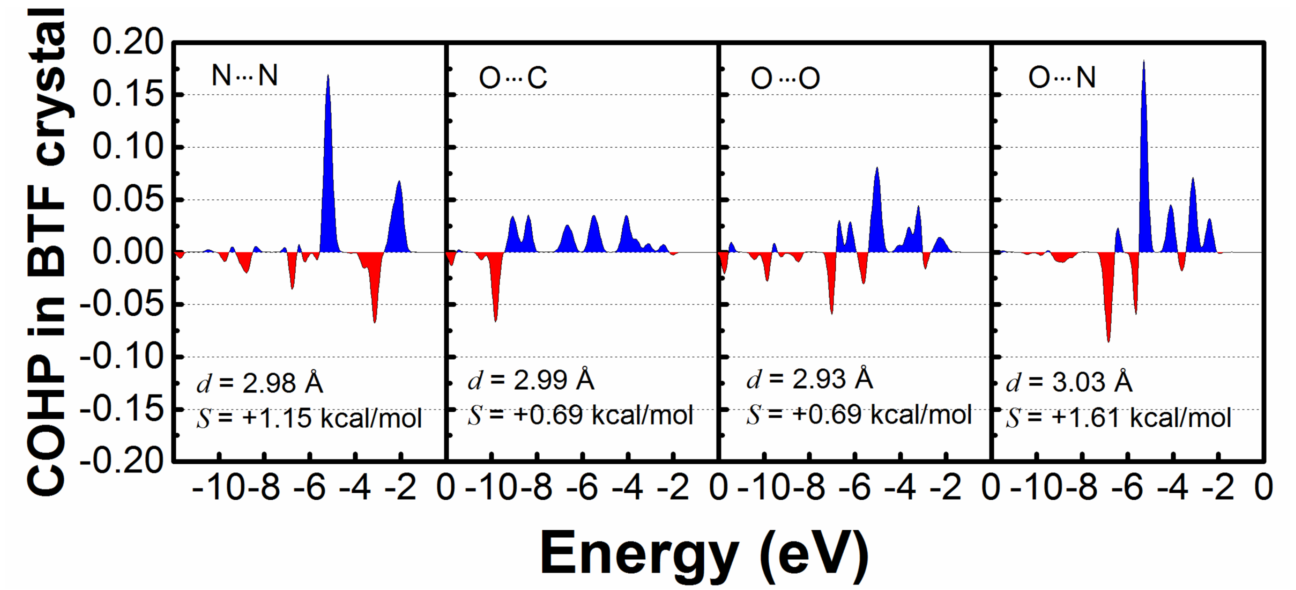


**Figure 2S** (Color online) Distance (*d*) and interacting strength (*S*) of the four kinds of inter-lone-pair interactions. All the inter-lone-pair interactions are antibonding and are mutually exclusive.

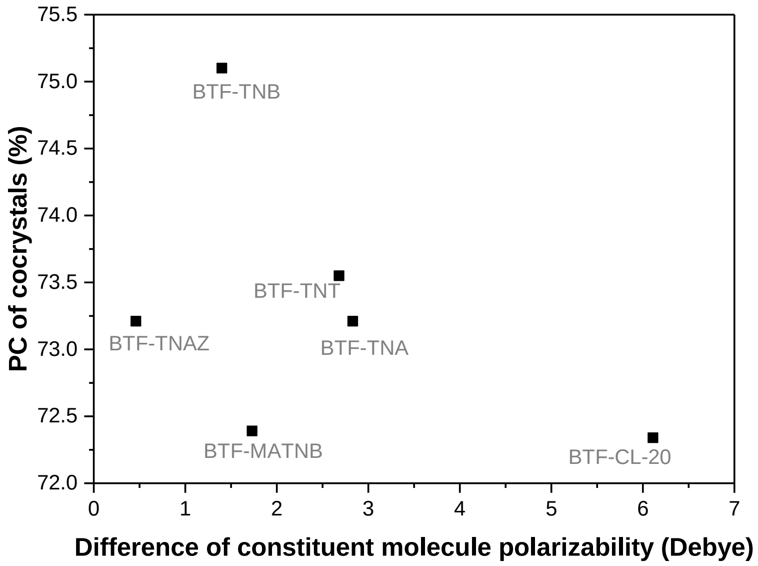


**Figure 3S** Packing coefficient of cocrystals along the difference of constituent molecule polarizability. Similar polarizability generally increases the solubility of two types of ingredient molecules.

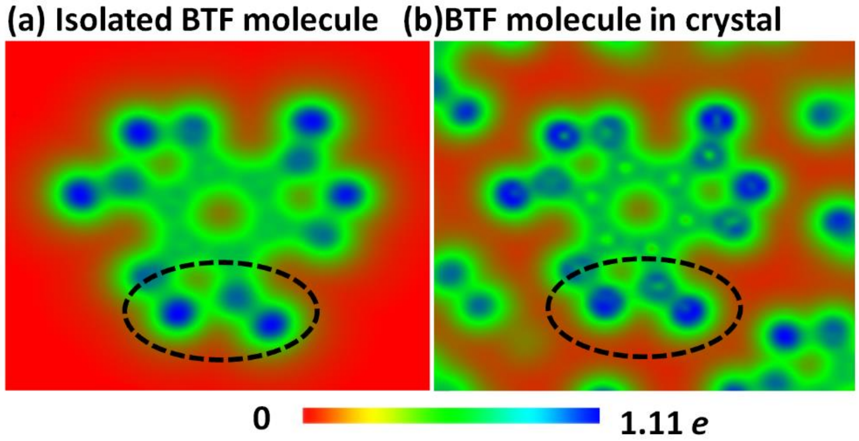


**Figure 4S** (Color online) Charge density aggregation at the trigger linkages (for chemical reaction initiation) of BTF crystal, indicating the improved sensitivity of the solid compared to its gas counterpart.

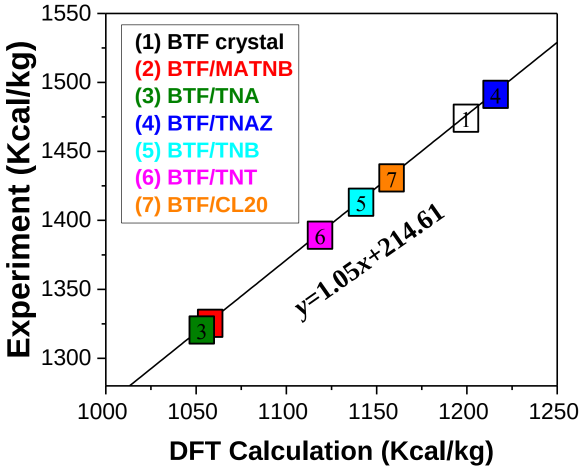


**Figure 5S** (Color online) Heat of explosion of all seven systems corrected by experiments.

**Table S1** Bond lengths (*d*, in Å) and interacting energy (*S*, in *kcal/mol*) for all the covalent bonds of an isolated BTF molecule and a BTF molecule in crystal. The trigger linkages for chemical reaction initiation are marked by the bold.

| | | | | | | | | | | | | |
|---|---|---|---|---|---|---|---|---|---|---|---|---|
| Oxofurazan ring 1 | **C-N** | | **N-O** | | **O-N$^+$** | | **N$^+$-O$^-$** | | **C-N$^+$** | | | |
| | *S* | *d* | *S* | *d* | ***S*** | | *S* | *d* | *S* | *d* | | |
| Crystal | -145.36 | 1.33 | -81.65 | 1.38 | **-46.23** | **1.61** | -148.81 | 1.24 | -145.36 | 1.34 | | |
| Isolated | -147.66 | 1.34 | -83.95 | 1.37 | **-42.09** | **1.66** | -145.13 | 1.24 | -147.66 | 1.34 | | |
| Oxofurazan ring 2 | C-N | | N-O | | **O-N$^+$** | | N$^+$-O$^-$ | | C-N$^+$ | | | |
| | *S* | *d* | *S* | *d* | ***S*** | ***d*** | *S* | *d* | *S* | *d* | | |
| Crystal | -146.74 | 1.34 | -83.26 | 1.38 | **-44.62** | **1.63** | -145.13 | 1.24 | -144.67 | 1.34 | | |
| Isolated | -147.66 | 1.34 | -83.95 | 1.37 | **-42.09** | **1.66** | -145.13 | 1.24 | -147.66 | 1.34 | | |
| Oxofurazan ring 3 | C-N | | N-O | | **O-N$^+$** | | N$^+$-O$^-$ | | C-N$^+$ | | | |
| | *S* | *d* | *S* | *d* | ***S*** | ***d*** | *S* | *d* | *S* | *d* | | |
| Crystal | -146.28 | 1.34 | -81.42 | 1.38 | **-45.31** | **1.61** | -145.13 | 1.24 | -145.59 | 1.34 | | |
| Isolated | -147.66 | 1.34 | -83.95 | 1.37 | **-42.09** | **1.66** | -145.13 | 1.24 | -147.66 | 1.34 | | |
| Benzene ring | C-C-1 | | C-C-2 | | C-C-3 | | C-C-4 | | C-C-5 | | C-C-6 | |
| | *S* | *d* | *S* | *d* | *S* | *d* | *S* | *d* | *S* | *d* | *S* | *d* |
| Crystal | -127.42 | 1.43 | -138.23 | 1.43 | -126.73 | 1.43 | -138.46 | 1.43 | -128.57 | 1.43 | -137.54 | 1.43 |
| Isolated | -129.72 | 1.43 | -139.38 | 1.43 | -129.72 | 1.43 | -139.38 | 1.43 | -129.72 | 1.43 | -139.38 | 1.43 |

**Table S2** Changes of interacting energies (*$\Delta S$*, in *kcal/mol*) for all the covalent bonds (intramolecular contacts) of the non-equivalent molecules of the BTF cocrystals. The corresponding values in the isolated molecules are taken as references and the values are set to zero. The trigger linkages for chemical reaction initiation are marked by the bold. *The symbols in Table S2 correspond to the atomic label of the provided supplementary xyz files.*

| | **BTF/MATNB** | | **BTF/TNA** | | **BTF/TNAZ** | | **BTF/TNB** | | **BTF/TNT** | | **BTF/CL-20** | |
|---|---|---|---|---|---|---|---|---|---|---|---|---|
| | Type | $\Delta S$ | Type | $\Delta S$ | Type | $\Delta S$ | Type | $\Delta S$ | Type | $\Delta S$ | Type | $\Delta S$ |
| Coformer molecule | H1-N14 | +2.53 | H1-N11 | +1.61 | H1-C5 | +2.99 | H1-C5 | +1.15 | H1-C8 | 0.00 | H1-C7 | +3.45 |
| | H2-C9 | -0.46 | H2-N11 | +1.38 | H2-C5 | +2.07 | H2-C7 | +0.46 | H2-C10 | -0.23 | H2-C8 | +3.22 |
| | H3-C11 | -1.84 | H3-C7 | +0.00 | H3-C7 | +2.30 | H3-C9 | +2.07 | H3-C12 | +3.45 | H3-C9 | +2.76 |
| | H4-C13 | +2.99 | H4-C9 | +0.00 | H4-C7 | +1.61 | C4-C5 | +2.76 | H4-C2 | +2.30 | H4-C10 | +3.22 |
| | H5-C13 | -0.46 | C5-C6 | +3.45 | C5-C6 | +1.84 | C5-C6 | +3.22 | H5-C12 | +2.76 | H5-C11 | +2.76 |
| | H6-C13 | +2.53 | C6-C7 | +0.92 | C6-C7 | +0.46 | C6-C7 | +1.61 | C6-C7 | +3.91 | H6-C12 | +3.68 |
| | C7-C8 | +0.69 | C7-C8 | +1.84 | C7-N8 | -1.61 | C7-C8 | +1.15 | C7-C8 | +0.46 | C7-C8 | 0.00 |
| | C8-C9 | -4.14 | C8-C9 | +2.76 | N8-C5 | -1.84 | C8-C9 | +2.99 | C8-C9 | +2.30 | C9-C12 | +0.92 |
| | C9-C10 | -2.53 | C9-C10 | +2.07 | C6-N10 | -2.07 | C9-C4 | +2.76 | C9-C10 | +1.61 | C10-C11 | 0.00 |
| | C10-C11 | -1.84 | C10-C5 | +3.45 | C6-N11 | -1.84 | C4-N10 | -2.07 | C10-C11 | +0.46 | N13-C7 | -1.38 |
| | C11-C12 | -2.07 | C5-N11 | -0.92 | N8-N9 | -10.35 | C8-N12 | -2.76 | C11-C6 | +2.76 | N13-C10 | +2.53 |
| | C12-C7 | +3.22 | C6-N12 | -2.53 | N9-O12 | +1.38 | C6-N11 | -2.07 | C7-N13 | -3.45 | N14-C8 | +2.76 |
| | C7-N14 | -5.75 | C8-N13 | -3.45 | N9-O13 | 0.00 | N10-O13 | -0.46 | C9-N14 | -4.83 | N14-C11 | +0.69 |
| | **C8-N15** | **-8.74** | C10-N14 | -2.30 | N10-O14 | +1.84 | N10-O14 | +0.92 | C11-N15 | -2.53 | N15-C8 | +0.46 |
| | **C10-N16** | **-5.06** | N12-O15 | -0.92 | N10-O15 | -1.15 | N11-O15 | -0.46 | N13-O16 | -0.69 | N15-C9 | -0.23 |
| | **C12-N17** | **-10.81** | N12-O16 | +2.30 | N11-O16 | -2.07 | N11-O16 | +2.30 | N13-O17 | +1.15 | N16-C7 | +1.61 |
| | O18-N15 | -3.22 | N13-O17 | +1.61 | N11-O17 | 0.00 | N12-O17 | +2.30 | N14-O18 | +1.84 | N16-C9 | +1.38 |
| | O19-N15 | +2.53 | N13-O18 | -0.46 | | | N12-O18 | +0.69 | N14-O19 | +1.15 | N17-C10 | 0.00 |
| | O20-N16 | -3.68 | N14-O19 | +1.84 | | | | | N15-O20 | +1.61 | N17-C12 | -1.38 |
| | O21-N16 | -5.75 | N14-O20 | -0.46 | | | | | N15-O21 | -2.07 | N18-C11 | +0.46 |
| | O22-N17 | -0.46 | | | | | | | | | N18-C12 | +0.23 |
| | O23-N17 | -2.76 | | | | | | | | | N19-N13 | -0.23 |
| | | | | | | | | | | | N20-N14 | -3.91 |
| | | | | | | | | | | | N21-N15 | -6.67 |
| | | | | | | | | | | | N22-N16 | -13.11 |
| | | | | | | | | | | | N23-N17 | -5.98 |
| | | | | | | | | | | | N24-N18 | -12.88 |
| | | | | | | | | | | | O25-N19 | +1.61 |
| | | | | | | | | | | | O26-N19 | +4.14 |
| | | | | | | | | | | | O27-N20 | +1.84 |
| | | | | | | | | | | | O28-N20 | +2.30 |
| | | | | | | | | | | | O29-N21 | +1.38 |
| | | | | | | | | | | | O30-N21 | -1.15 |

| | | | | | | | | | | | | |
|---|---|---|---|---|---|---|---|---|---|---|---|---|
| | | | | | | | | | | | O31-N22 | +2.53 |
| | | | | | | | | | | | O32-N22 | +2.30 |
| | | | | | | | | | | | O33-N23 | +0.46 |
| | | | | | | | | | | | O34-N23 | +0.46 |
| | | | | | | | | | | | O35-N24 | +4.37 |
| | | | | | | | | | | | O36-N24 | +0.69 |
| | Type | *ΔS* | *ΔS* | | *ΔS* | | *ΔS* | | *ΔS* | | *ΔS* | |
| BTF molecule | C3-N9 | -3.45 | +0.46 | | +1.61 | | +1.15 | | +0.46 | | +2.53 | |
| | N9-O15 | +0.46 | +1.15 | | +1.38 | | +0.46 | | -1.38 | | +2.76 | |
| | **O15-N8** | **-7.13** | **-2.53** | | **-1.38** | | **-2.53** | | **-1.15** | | **-1.38** | |
| | N8-O14 | -0.69 | +0.69 | | +1.38 | | -1.84 | | -2.30 | | +1.38 | |
| | N8-C2 | -11.04 | +0.69 | | +0.92 | | +1.84 | | +0.92 | | +2.76 | |
| | C5-N11 | -3.45 | +0.92 | | +1.61 | | +0.69 | | +0.23 | | +3.68 | |
| | N11-O17 | 0.00 | +4.37 | | +1.15 | | +3.91 | | +2.53 | | +5.98 | |
| | **O17-N10** | **-3.91** | **-5.98** | | **-1.38** | | **-5.75** | | **-3.22** | | **-6.44** | |
| | N10-O16 | -8.28 | -2.07 | | 0.00 | | -0.92 | | -0.23 | | +0.46 | |
| | N10-C4 | -1.15 | -2.76 | | +0.69 | | -3.45 | | +0.69 | | +1.84 | |
| | C1-N7 | -5.06 | +1.38 | | +0.69 | | +0.92 | | +0.46 | | +2.07 | |
| | N7-O13 | -0.46 | +3.45 | | +2.07 | | +1.61 | | +5.29 | | +4.83 | |
| | **O13-N12** | **-3.22** | **-3.91** | | **-2.07** | | **-2.76** | | **-5.98** | | **-2.99** | |
| | N12-O18 | +2.53 | +2.07 | | 0.00 | | +0.69 | | -2.30 | | +0.46 | |
| | N12-C6 | -6.21 | +1.15 | | +0.92 | | +1.38 | | -3.22 | | +4.60 | |
| | C4-C3 | -0.46 | +1.38 | | +1.61 | | +0.92 | | +0.69 | | +2.76 | |
| | C3-C2 | -3.22 | +0.92 | | +0.69 | | +0.92 | | +0.46 | | +2.99 | |
| | C2-C1 | +1.38 | +0.92 | | +1.38 | | +1.15 | | +1.15 | | +2.99 | |
| | C1-C6 | -3.68 | +0.69 | | +0.69 | | +1.15 | | +0.69 | | +1.84 | |
| | C6-C5 | +2.07 | +0.69 | | +0.92 | | 0.00 | | +0.23 | | +2.99 | |
| | C5-C4 | -2.07 | +0.92 | | +0.69 | | +1.15 | | +0.92 | | +2.53 | |

1 CrystalExplorer (Version 3.1) (University of Western Australia, 2012).
